# Quality of life after off-pump vs on-pump coronary artery bypass grafting. A prospective randomized single center study

**DOI:** 10.1186/1749-8090-8-S1-O203

**Published:** 2013-09-11

**Authors:** T Sakellaridis, N Kontodimopoulos, M Argiriou, M Kolokotroni, J Kokotsakis, V Patris, D Niakas, C Charitos

**Affiliations:** 1 2nd Cardiac Surgery Department, “Evangelismos” Hospital, Athens, Greece; 2 Hellenic Open University, Patra, Greece

## Background

CABG is an invasive method aiming to modify the natural course of the disease, to relief symptoms from the patients and to extend and improve patient’s life. The aim of this study is to measure and compare the quality of life of patients who have undergone conventional CABG and off-pump CABG.

## Methods

A total of 179 patients, who underwent CABG, within an eight month period from September 2011, took part in this prospective study. For the information acquisition, the General Health Evaluation questionnaires SF-36 (Short Form Health Survey), SF-6D, EQ-5D, 15D and the disease specific questionnaire Coronary Revascularization Outcome Questionnaire (CROQ) were used prior surgery and three months after. Age, gender, body mass index, smoking habit and Euroscore were recorded.

## Results

The two groups were comparable in terms of age, gender, body mass index, smoking habit. The results of the research showed a clear improvement in health-related quality of life of patients in both groups who underwent CABG, as compared to that prior to the intervention. But there were no statistically significant differences between the group that underwent conventional CABG and the group that underwent off-pump CABG, which is consistent with the existing literature. The index of SF-6D utility is highly correlated with CROQ when compared with the other utilities indexes and we propose its use in patients with coronary artery disease.

## Conclusion

CABG modifies and improves the clinical presentation and patient’s quality of life, with no difference in health-related quality of life between off-pump and conventional CABG.

